# Global Research on Nursing Work Environment and Structural Empowerment: Trends and Thematic Evolution in Leadership and Workforce Outcomes—A Bibliometric Analysis

**DOI:** 10.1155/jonm/6020647

**Published:** 2026-09-14

**Authors:** Xin Su, Mengnan Liu, Yan Jiang, Han He, Haobo Wang, Xinyi He, Xinyi Fan, Yanqi Wu, Gang Luo, Jinwen Wu

**Affiliations:** ^1^ The Affiliated Hospital, Southwest Medical University, Luzhou Sichuan, 646000, China, scu.edu.cn; ^2^ The Affiliated Traditional Chinese Medicine Hospital, Southwest Medical University, Luzhou Sichuan, 646000, China, scu.edu.cn; ^3^ School of Basic Medical Sciences, Southwest Medical University, Luzhou Sichuan, 646000, China, scu.edu.cn; ^4^ School of Nursing, Southwest Medical University, Luzhou Sichuan, 646000, China, scu.edu.cn; ^5^ School of Rehabilitation Medicine, Southwest Medical University, Luzhou Sichuan, 646000, China, scu.edu.cn

**Keywords:** bibliometric analysis, leadership, nursing work environment, structural empowerment, workforce outcomes

## Abstract

**Background:**

Nursing work environment (NWE) and structural empowerment (SE) are closely related to nurse well‐being, retention, leadership, care quality, and patient safety. However, their intellectual structure, thematic evolution, and policy relevance remain insufficiently synthesized amid global nursing workforce shortages.

**Aim:**

To map global research on NWE and SE and clarify its links with leadership development and nursing workforce outcomes.

**Methods:**

A bibliometric analysis was conducted using English‐language research articles retrieved from Web of Science Core Collection and Scopus. Publication trends, leading contributors, collaboration patterns, co‐cited references, keyword clusters, thematic evolution, and emerging topics were examined.

**Results:**

A total of 1470 publications from 2007 to 2026 were included. Annual output increased steadily, particularly after 2019. The United States, China, and Canada were leading contributors, and the University of Pennsylvania was the most productive institution. Influential scholarship connected practice environments, empowerment, leadership, burnout, retention, care quality, and patient safety. The field has shifted from describing adverse outcomes to explaining how organizational conditions and leadership support sustainable nursing workforces.

**Discussion:**

The findings indicate a transition toward mechanism‐oriented research explaining how SE and leadership connect organizational conditions with nurse well‐being, retention, and care quality. This highlights the need to treat empowering practice environments as nursing management and health policy priorities.

**Conclusion:**

Research is moving toward understanding how empowering work environments and leadership practices sustain nurses and improve care. Stronger longitudinal and intervention evidence is needed.

**Implications for Nursing Management and Policy:**

Workforce policy should prioritize organizational reforms that create empowering practice environments, support leadership development, and sustain a stable nursing workforce alongside measures to increase nurse supply.

## 1. Introduction

Nurses constitute the largest professional group in the global health workforce and are central to health system functioning, care quality, and patient safety. The World Health Organization estimates that there are approximately 29 million nurses and 2.2 million midwives worldwide; by 2030, however, the global shortage is projected to reach 4.5 million nurses and 310,000 midwives [1]. This shortage is not only a matter of workforce supply. It is also linked to turnover, occupational burnout, job dissatisfaction, and declining career sustainability [2].

In this context, the nursing work environment (NWE) has become a key policy and management concern. Evidence from multinational hospital studies indicates that the NWE and patient‐to‐nurse ratios are important organizational factors associated with nurses’ professional outcomes and patients’ care experiences [3]. Poor work environments are commonly associated with higher burnout, job dissatisfaction, and intention to leave, as well as lower care quality and patient satisfaction [4, 5]. Strategies to address the nursing workforce crisis therefore need to combine recruitment and education with sustained efforts to improve practice environments, strengthen organizational support, and promote nurse retention.

### 1.1. Theoretical Framework: Kanter’s Structural Empowerment Theory

Structural empowerment (SE) offers a theoretical lens for understanding why organizational conditions matter for workforce outcomes. Kanter’s Structural Empowerment Theory argues that power within organizations is not derived only from hierarchical position, but also from access to opportunity, information, support, resources, and formal and informal power [6, 7]. Opportunity refers to possibilities for professional growth and development; information refers to access to organizational knowledge needed to perform work effectively; support refers to feedback, guidance, and assistance from supervisors and colleagues; resources refer to adequate time, staffing, materials, and equipment; formal power reflects flexibility, visibility, and decision‐making discretion; and informal power reflects relationships and networks within and outside the organization.

Applied to nursing, SE refers to nurses’ access to organizational resources, institutional support, professional development opportunities, and participation in decision‐making [8]. Adequate SE can support professional autonomy, work engagement, and organizational influence, whereas limited empowerment may contribute to powerlessness, career stagnation, and organizational alienation [9]. Related nursing management frameworks, including practice environment models, shared governance, professional autonomy, and leadership theories, further suggest that empowering organizational conditions are translated into nursing practice through leadership, resource allocation, participation in decision‐making, and supportive team climates.

Leadership is therefore a key pathway through which the NWE and SE are translated into daily practice [10]. SE is not determined solely by individual nurses′ initiative; it is shaped by the conditions created by nursing leaders and managers through resource allocation, information sharing, delegation, decision‐making support, and team climate [11]. Previous studies have shown that transformational and authentic leadership are associated with job satisfaction, work performance, and patient safety through workplace empowerment [12]. Leadership therefore connects organizational conditions with nurse well‐being, nurse retention, work performance, and care quality.

Although research on these topics has expanded, the evidence remains dispersed across studies of the work environment, SE, leadership, and individual workforce outcomes. The broader knowledge structure linking these concepts has not been fully clarified. A bibliometric approach can identify influential contributors, intellectual foundations, research hotspots, and emerging themes, thereby showing how the field has developed and where nursing management and policy debates are moving.

Unlike reviews that examine isolated relationships between work environment, empowerment, leadership, or nurse outcomes, this study maps these concepts within a unified bibliometric framework. Specifically, we analyzed global research trends in NWE and SE, identified the knowledge base and thematic evolution of the field, and examined how NWE and SE are connected with leadership and nursing workforce outcomes. The findings are intended to inform future nursing management research, empowerment‐oriented practice, and organizational policy reform.

Accordingly, this study addressed the following research questions:

1. What are the global publication trends and major contributors in research on NWE and SE?
2. What are the main countries, institutions, authors, journals, and collaborative networks shaping this field?
3. What are the key intellectual foundations and thematic clusters identified through co‐citation and keyword analyses?
4. How have research themes evolved over time, particularly in relation to leadership, nursing workforce outcomes, and policy‐relevant organizational reform?

## 2. Methods

### 2.1. Data Acquisition and Search Strategy

A systematic literature search was conducted in the Web of Science Core Collection (WoSCC) and Scopus to identify publications related to the NWE and SE. The final retrieval was completed on March 31, 2026, and records from 2026 therefore represented partial‐year or Early Access publications. Because the earliest eligible record in the final dataset was published in 2007, this year was used as the starting point for the bibliometric analysis. Publication trend analysis was based on complete calendar years from 2007 to 2025, and 2026 records were reported separately rather than interpreted as part of the annual growth trend.

The search strategy combined terms related to nurses, work environment, SE, autonomy, and shared governance. In WoSCC, the search was performed using the topic field: TS = (nurs∗ AND (“work environment” OR “practice environment” OR “nursing work environment” OR “practice environment scale” OR “structural empowerment” OR autonomy OR “shared governance”)). The search strategy was adapted for Scopus using the TITLE‐ABS‐KEY field. The complete search strategies for WoSCC and Scopus, including field tags, Boolean operators, document type and language limits, and the search date, are provided in Supporting Table S1.

**FIGURE 1:**
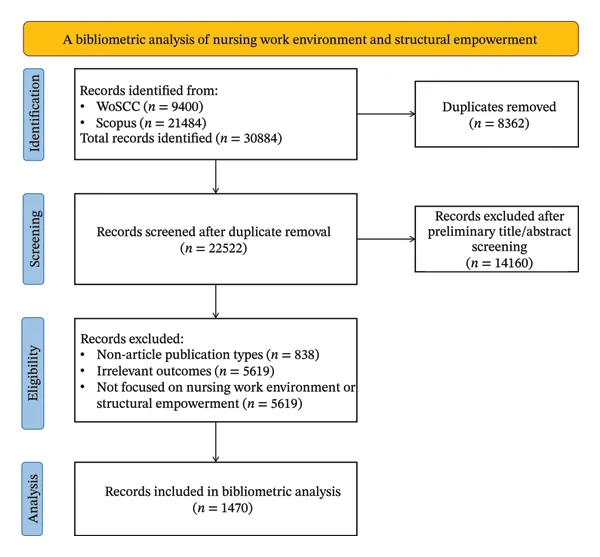
Flowchart of the bibliometric analysis of nursing work environment and structural empowerment research.

The search strategy was developed based on the study aim, the two core constructs of NWE and SE, and related organizational concepts identified from foundational and recent nursing management literature. Preliminary searches were conducted to refine the keywords and balance sensitivity and specificity. Although this study focused primarily on NWE and SE, the terms “autonomy” and “shared governance” were included because they represent important organizational dimensions closely related to nurses’ practice environments, empowerment structures, and participation in decision‐making. These terms were not searched independently but were combined with “nurs∗” to restrict retrieval to nursing‐related literature. Leadership and nursing workforce outcomes were not used as mandatory search concepts; rather, they were examined as thematic pathways emerging from the retrieved literature. The detailed retrieval and screening process is shown in the PRISMA 2020‐style flow diagram in Figure 1.

### 2.2. Inclusion and Exclusion Criteria

Publications were included if they met the following criteria: (1) the study was related to NWE, SE, autonomy, shared governance, or closely related organizational conditions in nursing; (2) the publication was an English‐language article; (3) full bibliographic information and cited references were available; and (4) the publication was indexed in WoSCC or Scopus before the retrieval date. Early access articles were retained if they met these criteria. The document type was limited to articles because original research articles provide more comparable empirical evidence and reduce heterogeneity introduced by reviews, editorials, letters, conference papers, book chapters, and other non‐original publication types.

After duplicate removal, 22,522 records remained for screening. Among these, 838 records were excluded because they were non‐article publication types. During preliminary title and abstract screening, 14,160 records were excluded because they were outside the conceptual scope of nursing organizational research, NWE, SE, leadership, or nursing workforce outcomes. A further 5619 records were excluded as “irrelevant outcomes” because they did not address nurse workforce, leadership, care quality, patient safety, or organizational outcomes. Finally, 435 records were excluded as “not focused on nursing work environment or structural empowerment” because nursing organizational conditions, empowerment, autonomy, shared governance, or closely related constructs were not central to the study. After screening and eligibility assessment, 1470 records were included in the final bibliometric analysis.

### 2.3. Data Merging, Bibliometric Parameters, and Data Cleaning

Records from WoSCC and Scopus were merged and harmonized before analysis. Duplicate records were identified primarily by DOI. For records without DOI or with inconsistent DOI formats, title, first author, and publication year were used for further checking. After duplicate removal, bibliographic fields, including author names, institutional names, country or region names, journal titles, keywords, and cited references, were checked and standardized where identifiable variations were detected.

For author analysis, author names were standardized by checking full names, initials, and common abbreviated forms where identifiable. Obvious name variants were merged when they referred to the same author based on co‐author information, affiliation, publication title, and research topic. Potentially ambiguous author names were not merged unless there was sufficient evidence to confirm that they referred to the same individual. For cited references, variations in cited author name, publication year, source title, volume, page number, and DOI were checked to reduce the possibility that the same reference appeared as multiple nodes in co‐citation networks. For keyword, institution, and journal analyses, plural forms, spelling variants, abbreviations, and synonymous expressions were standardized where appropriate to reduce node fragmentation.

Citation indicators, including total citations, average citations, and H‐index, were calculated from the harmonized dataset after deduplication. Citation counts from WoSCC and Scopus were not summed to avoid double counting. For records indexed in both databases, the citation count from the retained master record was used; for records indexed in only one database, the citation count from that database was retained. The H‐index reported in this study was calculated only from publications included in the final dataset and should therefore be interpreted as a field‐specific indicator rather than as the overall H‐index of each country, institution, author, or journal.

The list of included records with DOI and title information is provided in Supporting Table S2. The key parameters used in CiteSpace and VOSviewer analyses, including network type, node type, time slicing, threshold selection, counting method, normalization method, pruning algorithm, and clustering or labeling algorithm, are summarized in Supporting Table S3.

### 2.4. Data Analysis

Microsoft Excel 2021, VOSviewer (Version 1.6.19), SCImago Graphica (Version 1.0.42), CiteSpace (Version 7.0.R1), and an online bibliometric platform (https://bibliometric.com/app) were used for bibliometric analysis and visualization.

Microsoft Excel 2021 was used to summarize annual publication trends and descriptive bibliometric indicators. SCImago Graphica was used to visualize the geographic distribution of publications, and the online bibliometric platform was used to visualize international collaboration among countries. VOSviewer was used to construct collaboration, co‐occurrence, and co‐citation networks, including institution collaboration, author collaboration, author co‐citation, journal and co‐cited journal analyses, and keyword co‐occurrence analysis [13]. CiteSpace was used to perform dual‐map overlay analysis, reference co‐citation clustering, timeline visualization, keyword clustering, and citation burst detection for references and keywords [14].

In network maps, nodes represented bibliometric items, node size reflected frequency or weight, links represented collaboration, co‐occurrence, or co‐citation relationships, and link strength reflected the intensity of the relationship. Detailed bibliometric parameters are summarized in Supporting Table S3.

Ethics approval and informed consent were not required because this bibliometric review used publicly available bibliographic records and did not involve human participants, human tissue, identifiable personal data, or animal experiments.

## 3. Results

### 3.1. Global Publication Distribution

**FIGURE 2:**
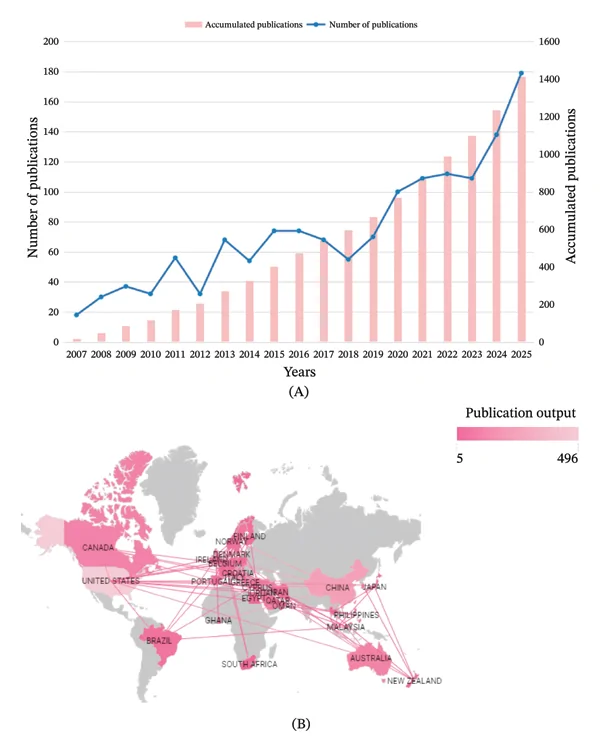
Global publication distribution and annual trend. (A) Annual publication trend based on complete calendar years from 2007 to 2025. Records from 2026 were partial‐year or early access publications and were not included in trend interpretation. (B) Geographic distribution of publications by country or region. Darker shading indicates a greater number of publications.

A total of 1470 publications on NWE and SE were included. The earliest article was published in 2007. Annual publication trends were analyzed using complete calendar years from 2007 to 2025. During this period, annual output showed an overall upward trend (Figure 2A). Publications remained fewer than 40 per year during 2007–2010, increased with fluctuations after 2013, and rose more rapidly after 2019. Annual output exceeded 100 publications after 2020 and reached its highest level in 2025. Records from 2026 were retrieved only up to March 31, 2026 and therefore represented partial‐year or early access publications. These records were reported separately and were not used to interpret the overall publication growth trend. Publications were distributed across 78 countries or regions, with major contributions from Europe, North America, and East Asia (Figure 2B).

### 3.2. Contributions of Countries and Institutions

**FIGURE 3:**
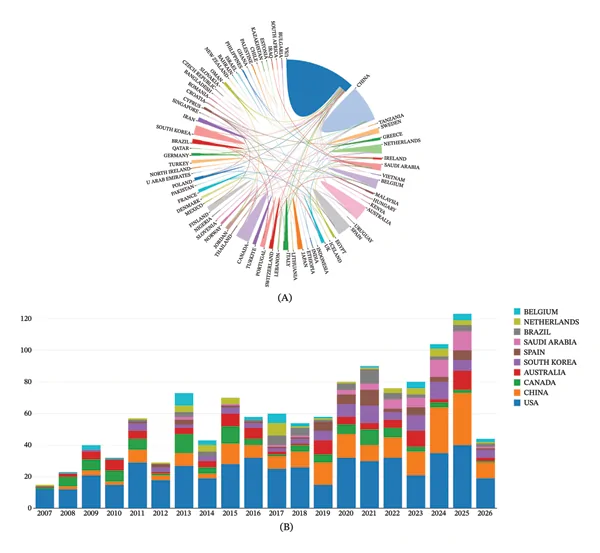
Country contributions and collaboration. (A) Country collaboration network. (B) Annual publication distribution of the top 10 contributing countries.

The top 10 productive countries are shown in Table 1. The United States contributed the largest number of publications (*n* = 495, 33.67%), followed by China (*n* = 228, 15.51%), Canada (*n* = 105, 7.14%), Australia (*n* = 95, 6.46%), and South Korea (*n* = 95, 6.46%). The United States also had the highest total citations (18,218) and H‐index (67), while China ranked second in total citations (6485) and had an H‐index of 45. Canada had the highest average citation count among the top 10 countries (66.47). The country collaboration network placed the United States and China in central positions (Figure 3A), and the annual country distribution showed early dominance by the United States with a marked increase in China’s output during 2024–2025 (Figure 3B).

**TABLE 1:** Top 10 productive countries/regions.

Rank	Country	Publications	Total citations	Average citations	H‐index
1	United States	495 (33.67%)	18,218	36.80	67
2	China	228 (15.51%)	6485	28.44	45
3	Canada	105 (7.14%)	6979	66.47	52
4	Australia	95 (6.46%)	2885	30.37	29
5	South Korea	95 (6.46%)	2561	26.96	24
6	Turkey	57 (3.88%)	720	12.63	17
7	Saudi Arabia	54 (3.67%)	772	14.30	16
8	Spain	50 (3.40%)	1273	25.46	17
9	Belgium	46 (3.13%)	2635	57.28	26
10	Brazil	46 (3.13%)	885	19.24	18

**FIGURE 4:**
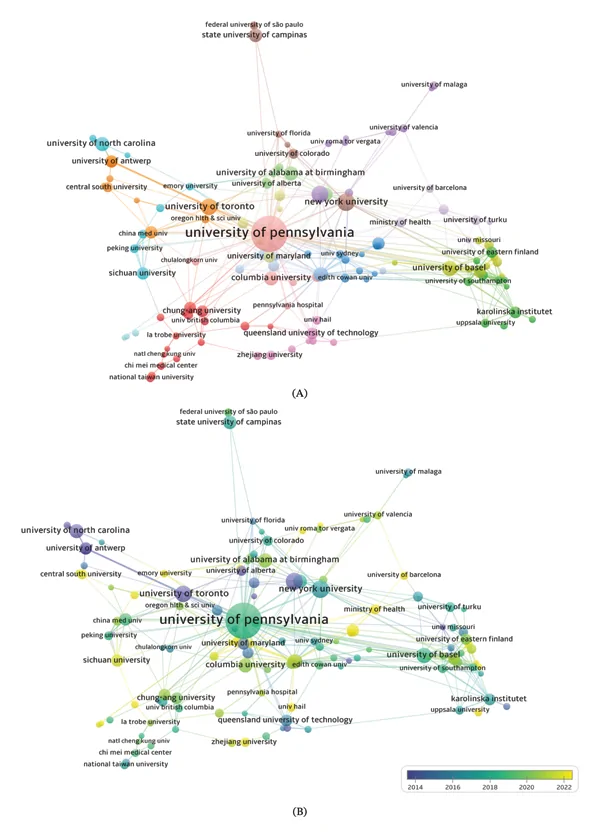
Institutional collaboration network. (A) Network visualization of institutional collaboration. (B) Overlay visualization of institutional collaboration. In panel (B), node color indicates the average publication year of each institution’s publications included in the network; the color scale reflects the range of average publication years among the displayed institutions.

At the institutional level, 2046 institutions contributed to this field. The top 10 institutions are listed in Table 2. The University of Pennsylvania was the most productive institution, with 96 publications (6.53%), and also ranked highly in total citations, average citations, and H‐index. New York University (*n* = 28, 1.90%), the University of Western Ontario (*n* = 26, 1.77%), and the University of Toronto (*n* = 25, 1.70%) also showed strong citation performance. The institutional collaboration network placed the University of Pennsylvania at the center (Figure 4A), while overlay visualization indicated earlier collaborations among North American universities and more recent participation from institutions in Asia and the Middle East, including Sichuan University, Zhejiang University, King Saud University, and Jordan University of Science and Technology (Figure 4B).

**TABLE 2:** Top 10 productive institutions.

Rank	Institution	Publications	Total citations	Average citations	H‐index
1	University of Pennsylvania	96 (6.53%)	5764	60.67	43
2	New York University	28 (1.90%)	917	32.75	19
3	The University of Western Ontario	26 (1.77%)	2756	106.00	25
4	University of Toronto	25 (1.70%)	2321	92.84	21
5	University of Basel	22 (1.50%)	1046	47.55	14
6	Case Western Reserve University	22 (1.50%)	590	26.82	14
7	Columbia University	20 (1.36%)	787	39.35	12
8	The University of Alabama at Birmingham	20 (1.36%)	375	18.75	15
9	University of North Carolina	19 (1.29%)	806	42.42	15
10	Jordan University of Science and Technology	19 (1.29%)	513	27.00	11

### 3.3. Contributions of Authors and Journals

**FIGURE 5:**
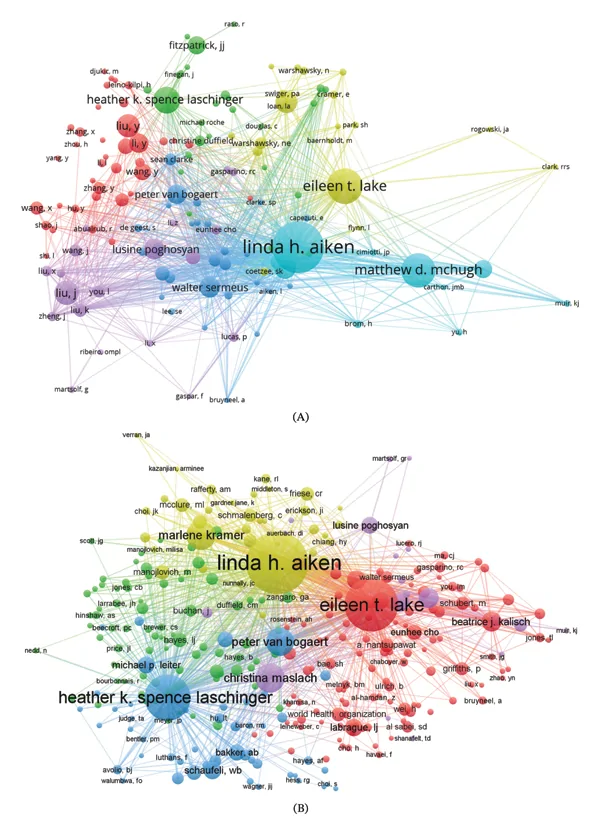
Author and co‐cited author networks. (A) Author collaboration network. (B) Co‐cited author network.

A total of 4897 authors contributed to NWE and SE research. Table 3 lists the top 10 most prolific authors. Linda H. Aiken was the most productive author, with 42 publications (2.86%), 4034 total citations, and an H‐index of 29. Eileen T. Lake, Matthew D. McHugh, Douglas M. Sloane, and Walter Sermeus were also among the leading authors (Figure 5A). Co‐cited author analysis showed a dense intellectual network involving Linda H. Aiken, Eileen T. Lake, Heather K. Spence Laschinger, Marlene Kramer, and Matthew D. McHugh (Figure 5B).

**TABLE 3:** Top 10 authors and co‐cited authors.

Rank	Most active authors					Most active co‐cited authors		
	Author	Publications	Total citations	Average citations	H‐index	Co‐cited author	Citations	Total link strength
1	Linda H. Aiken	42 (2.86%)	4034	94.05	29	Linda H. Aiken	1452	24,558
2	Eileen T. Lake	29 (1.97%)	1261	43.48	17	Eileen T. Lake	1109	16,223
3	Matthew D. McHugh	27 (1.84%)	967	35.81	13	Heather K. Spence Laschinger	853	13,936
4	Douglas M. Sloane	18 (1.22%)	2761	153.39	16	Vanaert Peter	356	6628
5	Walter Sermeus	17 (1.16%)	924	54.35	12	Christina Maslach	377	6289
6	Heather K. Spence Laschinger	17 (1.16%)	1880	110.59	22	Marlene Kramer	335	5667
7	Patricia A. Patrician	17 (1.16%)	367	21.59	12	Matthew D. McHugh	196	3635
8	Lusine Poghosyan	16 (1.09%)	908	56.75	14	Beatrice J. Kalisch	203	3420
9	Peter Van Bogaert	15 (1.02%)	1449	96.60	15	Michael P. Leiter	150	3223
10	Karen B. Lasater	15 (1.02%)	512	34.13	7	Ann Kutney‐Lee	182	3177

**FIGURE 6:**
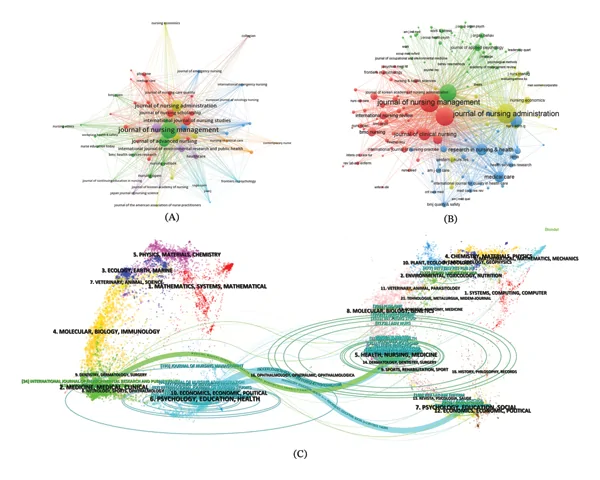
Journal distribution and citation pathways. (A) Network visualization of citing journals. (B) Network visualization of co‐cited journals. (C) Dual‐map overlay of journals. In panel (C), the left side represents the disciplinary distribution of citing journals, the right side represents the disciplinary distribution of cited journals, and the curved lines indicate citation pathways between disciplines.

Studies on NWE and SE were published in 220 journals. Table 4 lists the top 10 journals by publication output and their 2025 quartile rankings in the Journal Citation Reports. The *Journal of Nursing Management* was the most productive journal, publishing 193 articles (13.13%), followed by the *Journal of Nursing Administration* (*n* = 98, 6.67%) and the *Journal of Advanced Nursing* (*n* = 87, 5.92%) (Figure 6A). Journal co‐citation analysis showed that the *Journal of Nursing Management* had the highest co‐citation frequency, with 2872 citations and a total link strength of 87,729 (Figure 6B). The *Journal of Nursing Administration* and the *International Journal of Nursing Studies* were also located at the core of the co‐citation network. The dual‐map overlay provides an overview of interdisciplinary citation pathways (Figure 6C). In this map, the left side represents the disciplinary distribution of citing journals, the right side represents the disciplinary distribution of cited journals, and the curved lines indicate citation pathways between disciplines. The main pathways extended from the “Psychology, Education, Health” and “Economics, Economic, Political” fields to “Health, Nursing, Medicine,” suggesting that this field draws on multidisciplinary knowledge while remaining grounded in nursing and health sciences.

**TABLE 4:** Top 10 citing journals and co‐cited journals.

Rank	Most active journals				Most active co‐cited journals				
	Journal	Publications	IF (2025)	JCR (2025)	Journal	Citations	Total link strength	IF (2025)	JCR (2025)
1	*Journal of Nursing Management*	193 (13.13%)	4.4	Q1	*Journal of Nursing Management*	2872	87,729	4.4	Q1
2	*Journal of Nursing Administration*	98 (6.67%)	1.8	Q2	*Journal of Nursing Administration*	2675	75,118	1.8	Q2
3	*Journal of Advanced Nursing*	87 (5.92%)	3.8	Q1	*International Journal of Nursing Studies*	2521	79,479	8.7	Q1
4	*BMC Nursing*	71 (4.83%)	5.2	Q1	*Journal of Advanced Nursing*	2157	70,401	3.8	Q1
5	*International Journal of Nursing Studies*	65 (4.42%)	8.7	Q1	*Nursing Research*	1187	36,161	2.5	Q2
6	*Journal of Clinical Nursing*	56 (3.81%)	3.6	Q1	*Journal of Clinical Nursing*	1172	37,859	3.6	Q1
7	*Journal of Nursing Scholarship*	49 (3.33%)	3.9	Q1	*Research in Nursing & Health*	1108	33,621	2.8	Q1
8	*International Nursing Review*	35 (2.38%)	4.8	Q1	*Medical Care*	975	30,051	1.9	Q3
9	*International Journal of Environmental Research and Public Health*	34 (2.31%)	NA	NA	*Journal of Nursing Scholarship*	752	23,838	3.9	Q1
10	*Healthcare*	26 (1.77%)	3.4	Q2	*BMC Nursing*	692	22,099	5.2	Q1

### 3.4. Highly Cited Publications and Reference Analysis

Table 5 presents the top 10 most cited publications. The most cited article was “Effects of Nurse Staffing and Nurse Education on Patient Deaths in Hospitals with Different Nurse Work Environments” by Linda H. Aiken et al., with 677 citations. The second most cited article was “Nurses’ Intention to Leave Their Profession: A Cross Sectional Observational Study in 10 European Countries” by Maud M. Heinen et al., which examined work environment‐related factors associated with nurses’ intention to leave.

**TABLE 5:** Top 10 cited publications.

Rank	Title	Journal	Author	Citations	IF (2025)	JCR (2025)
1	Effects of Nurse Staffing and Nurse Education on Patient Deaths in Hospitals With Different Nurse Work Environments	*Medical Care*	Linda H. Aiken	677	1.9	Q3
2	Nurses′ Intention to Leave Their Profession: A Cross Sectional Observational Study in 10 European Countries	*International Journal of Nursing Studies*	Maud M. Heinen	377	8.7	Q1
3	Importance of Work Environments on Hospital Outcomes in Nine Countries	*International Journal for Quality in Health Care*	Linda H. Aiken	330	2.6	Q2
4	Nursing Staffing, Nursing Workload, the Work Environment and Patient Outcomes	*Applied Nursing Research*	Christine Duffield	329	2.2	Q2
5	Effect of Transformational Leadership on Job Satisfaction and Patient Safety Outcomes	*Nursing Outlook*	Sheila A. Boamah	289	3.8	Q1
6	Violence Toward Nurses, the Work Environment, and Patient Outcomes	*Journal of Nursing Scholarship*	Michael Roche	285	3.9	Q1
7	Authentic Leadership, Performance, and Job Satisfaction: the Mediating Role of Empowerment	*Journal of Advanced Nursing*	Carol A. Wong	280	3.8	Q1
8	Effects of Nurse Work Environment on Job Dissatisfaction, Burnout, Intention to Leave	*International Nursing Review*	A. Nantsupawat	267	4.8	Q1
9	How Nurses and Their Work Environment Affect Patient Experiences of the Quality of Care: A Qualitative Study	*BMC Health Services Research*	Renate Amm Kieft	263	3.3	Q2
10	Physician and Nurse Well‐Being and Preferred Interventions to Address Burnout in Hospital Practice: Factors Associated with Turnover, Outcomes, and Patient Safety	*JAMA Health Forum*	Linda H. Aiken	250	12.5	Q1

**FIGURE 7:**
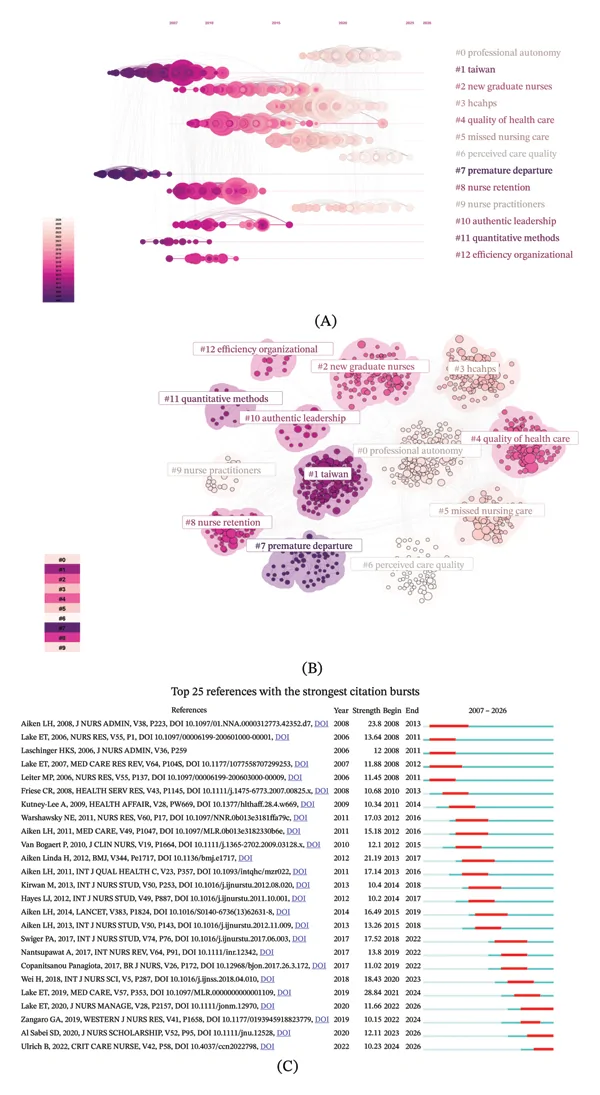
Reference co‐citation analysis and citation bursts. (A) Timeline view of reference co‐citation analysis. (B) Cluster view of reference co‐citation analysis. (C) Top 25 references with the strongest citation bursts.

Co‐cited reference analysis identified the key literature and intellectual foundations of the field (Table 6). Lake’s development of the Practice Environment Scale of the Nursing Work Index was the most frequently co‐cited reference, with 572 citations, followed by Aiken’s studies on the Revised Nursing Work Index, nurse staffing, burnout, and mortality. Co‐citation clusters and timeline views indicated a shift from foundational measurement studies toward leadership, empowerment, retention, and workforce sustainability (Figure 7A,B). Reference citation burst analysis identified 25 references with strong temporal influence from 2007 to 2026 (Figure 7C). Earlier bursts focused on work environment measurement, SE, burnout, job satisfaction, and nurse retention, whereas more recent bursts highlighted burnout, missed care, workforce well‐being, and the postpandemic work environment.

**TABLE 6:** Top 10 co‐cited references.

Rank	Title	Journal	Author	Citations	IF (2025)	JCR (2025)
1	Development of the Practice Environment Scale of the Nursing Work Index	*Research in Nursing & Health*	Eileen T. Lake	572	2.8	Q1
2	Measuring Organizational Traits of Hospitals: The Revised Nursing Work Index	*Nursing Research*	Linda H. Aiken	177	2.5	Q2
3	Hospital Nurse Staffing and Patient Mortality, Nurse Burnout, and Job Dissatisfaction	*JAMA*	Linda H. Aiken	177	65.4	Q1
4	Effects of Hospital Care Environment on Patient Mortality and Nurse Outcomes	*Journal of Nursing Administration*	Linda H. Aiken	171	1.8	Q3
5	Patient Safety, Satisfaction, and Quality of Hospital Care: Cross Sectional Surveys of Nurses and Patients in 12 Countries in Europe and the United States	*BMJ*	Linda H. Aiken	127	55.1	Q1
6	Global Use of the Practice Environment Scale of the Nursing Work Index	*Nursing Research*	Nora E. Warshawsky	113	2.5	Q2
7	Variations in Nursing Practice Environments: Relation to Staffing and Hospital Characteristics	*Nursing Research*	Eileen T. Lake	112	2.5	Q2
8	A Meta‐Analysis of the Associations Between the Nurse Work Environment in Hospitals and 4 Sets of Outcomes	*Medical Care*	Eileen T. Lake	110	1.9	Q3
9	Nurses′ Reports on Hospital Care in Five Countries	*Health Affairs*	Eileen T. Lake	108	6.7	Q1
10	The Nursing Practice Environment: Measurement and Evidence	*Medical Care Research and Review*	Eileen T. Lake	105	2.7	Q2

### 3.5. Keyword Analysis: Clusters, Bursts, and Thematic Evolution

**FIGURE 8:**
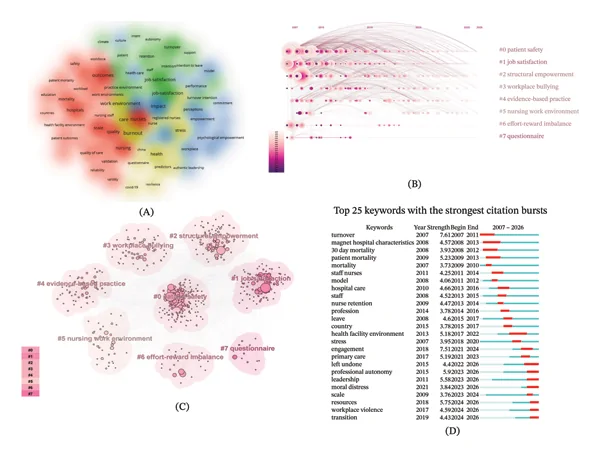
Keyword analysis and burst detection. (A) Keyword density map. (B) Timeline view of keyword analysis. (C) Cluster view of keyword analysis. (D) Top 25 keywords with the strongest citation bursts.

The top 20 keywords are listed in Table 7. The most frequent keyword was job satisfaction (430 occurrences), followed by work environment (364), burnout (285), stress (122), turnover (117), patient safety (114), and mortality (103). Keywords related to empowerment and leadership, including psychological empowerment, SE, leadership, authentic leadership, and transformational leadership, were also prominent. The keyword co‐occurrence and density maps showed interconnected themes involving practice environments, nurse well‐being and burnout, workforce retention and turnover, leadership and empowerment, and patient safety and care quality (Figure 8A,B).

**TABLE 7:** Top 20 most frequently occurring keywords.

Rank	Keyword	Occurrences
1	Job satisfaction	430
2	Work environment	364
3	Burnout	285
4	Stress	122
5	Turnover	117
6	Patient safety	114
7	Mortality	103
8	Perceptions	87
9	Leadership	64
10	Psychological empowerment	63
11	Structural empowerment	61
12	Work engagement	43
13	Intention to leave	42
14	Mental health	41
15	Organizational commitment	38
16	Nursing care	37
17	Authentic leadership	28
18	Left undone	26
19	Transformational leadership	24
20	Workplace violence	23

Keyword cluster analysis identified eight major thematic areas (Figure 8C). The patient safety cluster reflected studies linking NWEs with care quality, mortality, patient outcomes, and safety‐related indicators. The job satisfaction cluster represented nurse workforce outcomes, including job satisfaction, retention, turnover intention, organizational support, and workplace climate. The SE cluster captured the theoretical and mechanism‐oriented dimension of the field, including empowerment, psychological empowerment, resources, commitment, performance, and authentic leadership. The workplace bullying and effort–reward imbalance clusters reflected adverse psychosocial work conditions, stress, workload, and occupational health concerns. The evidence‐based practice cluster indicated attention to professional practice, education, research use, and quality improvement, whereas the NWE cluster represented the core conceptual domain of this study. The questionnaire cluster reflected the methodological foundation of the field, including scale development, validation, reliability, and survey‐based research.

Citation burst analysis showed a temporal shift in research priorities (Figure 8D). Earlier burst terms, including turnover, magnet hospital characteristics, 30‐day mortality, patient mortality, mortality, staff nurses, and hospital care, reflected attention to hospital work environments and measurable nurse or patient outcomes. More recent bursts, including engagement, professional autonomy, leadership, moral distress, resources, workplace violence, and transition, suggest increasing attention to empowerment mechanisms, organizational support, psychosocial work conditions, and workforce sustainability. These patterns indicate that the field has gradually shifted from describing associations between work environments and outcomes toward explaining the organizational mechanisms through which NWEs influence nurse well‐being, retention, care quality, and patient safety.

## 4. Discussion

This bibliometric analysis provides an overview of global research on NWE and SE from 2007 to 2026. Previous reviews have clarified specific aspects of this field, including work environment interventions [15], nurse and patient outcomes [16], measurement instruments [17], SE [18], leadership [19], and burnout‐related outcomes [20]. Building on this evidence, the present study maps the broader knowledge structure and thematic evolution of the field by identifying publication trends, collaboration networks, influential literature, keyword clusters, and burst terms. These findings show that the field has gradually moved from outcome‐oriented studies toward mechanism‐oriented research linking empowerment, leadership, autonomy, resources, and workforce sustainability.

### 4.1. General Information

The publication trajectory suggests that NWE and SE have moved from a relatively focused nursing management topic to a broader field concerned with workforce sustainability, care quality, and patient safety. This development mirrors the policy challenge outlined in the background: Increasing the number of nurses is necessary, but it is unlikely to be sufficient if nurses practice in environments that undermine retention and care quality. The early dominance of the United States and the central role of the University of Pennsylvania reflect long‐standing research programs on nursing practice environments, staffing, and patient outcomes. The rapid growth of publications from China in recent years suggests that nurse retention, burnout, and organizational support are also becoming increasingly visible in non‐Western health systems.

The concentration of influential publications in a limited number of countries, institutions, and journals has shaped the intellectual structure of the field. Core journals such as the *Journal of Nursing Management* and the *Journal of Nursing Administration* served as both major publication sources and highly co‐cited journals, anchoring the topic in nursing management and administration. The dual‐map overlay also indicates that the field draws on psychology, education, management, economics, and health policy, which is consistent with the organizational and policy relevance of NWE and SE research.

The author and co‐citation patterns point to complementary research traditions rather than a single line of inquiry. Aiken and Lake provided the empirical foundation for linking nursing practice environments, staffing, and patient outcomes, while Laschinger advanced the SE tradition by connecting access to resources, support, opportunity, leadership, and nurse work outcomes. McHugh and other scholars extended this evidence toward burnout, workforce outcomes, and care quality. Taken together, these knowledge communities support a mechanism‐oriented interpretation of the field: The NWE defines the organizational context, SE explains how resources and support become accessible to nurses, leadership activates these conditions in practice, and workforce outcomes indicate whether the system is sustainable.

### 4.2. Thematic Evolution of Research Hotspots and Emerging Topics

Based on the reference timeline, keyword clustering, keyword burst analysis, and qualitative interpretation of representative literature, the research frontiers can be summarized into four major themes. The keyword clusters indicate the main thematic domains of the field, whereas the burst keywords further show changes in research attention over time. Together, these results demonstrate a progression from measurement and outcome description toward mechanism‐oriented research on empowerment, leadership, and workforce sustainability.

#### 4.2.1. From Measurement of the NWE to Evidence on Patient and Workforce Outcomes

Early research in this field was strongly shaped by the development and application of standardized measures of nursing practice environments. Lake developed the Practice Environment Scale of the Nursing Work Index [21], which provided a widely used instrument for assessing organizational attributes such as nurse participation in hospital affairs, foundations for quality care, nurse manager support, staffing adequacy, and nurse–physician relations. This work was important because it transformed the NWE from a broad managerial concept into a measurable construct that could be compared across organizations and health systems.

Aiken and colleagues extended this measurement tradition by linking NWEs with nurse staffing, nurse education, patient mortality, burnout, job dissatisfaction, and care quality across hospital and multicountry settings. For example, their medical care study showed that the effects of nurse staffing and nurse education on patient deaths varied according to hospital work environments [22]. Their subsequent multicountry studies further showed that better work environments were associated with lower burnout, less job dissatisfaction, and more favorable patient safety and quality‐of‐care outcomes [23]. Other studies connected work environments with intention to leave, patient safety, missed care, and perceived care quality [24]. This evidence positions the NWE as more than a descriptive background condition; it is an organizational context closely linked with nurse retention, care quality, and patient safety.

#### 4.2.2. SE as a Mechanism Explaining Why Work Environments Matter

SE has become a key theoretical mechanism for explaining how organizational environments influence nurses’ professional experiences. Drawing on Kanter’s Structural Empowerment Theory, Laschinger and colleagues applied SE to nursing and showed that nurses’ access to information, resources, support, opportunities, and formal and informal power was associated with work satisfaction, organizational commitment, psychological empowerment, job strain, and burnout‐related outcomes [25, 26]. This line of research moved the field from asking whether work environments are associated with outcomes toward asking how organizational structures are translated into nurses’ daily work experiences.

The findings of the present bibliometric analysis support the continued relevance of Kanter’s theory in nursing management research. The co‐citation and keyword analyses showed that SE, professional autonomy, shared governance, leadership, job satisfaction, burnout, retention, care quality, and patient safety were closely connected within the knowledge structure of this field. This pattern is consistent with Kanter’s view that access to resources, information, support, opportunity, and formal and informal power shapes employees’ capacity to perform effectively and influence organizational outcomes.

This perspective is particularly relevant for interpreting the relationship between the NWE and workforce outcomes. Similar staffing levels or formal policies may be experienced differently across institutions if nurses differ in their access to resources, decision‐making opportunities, and managerial support. Empirical studies on workplace empowerment have linked SE with work satisfaction, psychological empowerment, work engagement, and organizational commitment [27]. SE therefore provides a conceptual bridge between the structural features of the work environment and nurses’ psychological, professional, and behavioral responses.

At the same time, the thematic evolution identified in this study suggests that the application of SE theory has expanded in contemporary nursing research. Earlier studies focused mainly on measuring practice environments and linking them with nurse and patient outcomes, whereas more recent studies increasingly examine leadership, professional autonomy, shared governance, missed care, workplace violence, resilience, and workforce sustainability. These emerging themes extend Kanter’s theory from an organizational empowerment framework toward a broader nursing workforce sustainability framework. In this expanded view, nurse leaders are not only managers of organizational resources but also key actors who translate organizational structures into empowering practice environments that support nurse well‐being, retention, care quality, and patient safety.

#### 4.2.3. Leadership as the Implementation Pathway From Empowerment to Practice Change

Recent studies increasingly position leadership as the pathway through which empowerment becomes actionable in clinical practice [28]. Boamah and colleagues found that transformational leadership was associated with nurses’ job satisfaction and patient safety outcomes, partly through workplace empowerment [10]. Wong and colleagues similarly showed that authentic leadership could improve job satisfaction and performance through empowerment‐related mechanisms [12]. These findings suggest that leadership should not be treated only as one dimension of the work environment; it is also an implementation process that shapes whether resources, autonomy, support, and opportunities are accessible to nurses.

This leadership pathway has direct implications for nursing management [19]. Supportive and empowering leaders can help translate organizational structures into a supportive practice climate, professional recognition, shared decision‐making, and team engagement [12]. Conversely, weak or unsupportive leadership may limit the benefits of otherwise favorable organizational conditions. Future research should examine leadership as a mediator, moderator, and implementation mechanism in the relationship between SE and nurse outcomes [10]. Intervention studies are especially needed to determine which leadership behaviors are associated with sustained improvements in burnout, nurse retention, work engagement, and patient safety.

#### 4.2.4. From Nurse Retention to Workforce Sustainability in the Postpandemic Context

The most recent research frontier has expanded from traditional retention indicators to the broader issue of nursing workforce sustainability [29]. Earlier highly cited studies established job satisfaction, burnout, turnover intention, and retention as central workforce outcomes [3, 30]. More recent keyword bursts and highly cited publications show increasing attention to resilience, missed nursing care, workplace violence, moral distress, mental health, professional autonomy, resources, and transition [31–33]. These topics suggest that workforce sustainability requires more than keeping nurses employed; it also requires conditions that support safe practice, professional identity, psychological well‐being, and high‐quality care.

The postpandemic literature makes this shift particularly clear. Studies conducted during and after COVID‐19 have shown that poor work environments continue to be associated with burnout, intention to leave, missed care, and reduced care quality under conditions of health system stress [32, 34, 35]. The central question is therefore not only whether nurses remain in the workforce, but under what organizational conditions they can practice safely, recover from cumulative stress, maintain professional identity, and remain engaged in care delivery. Future studies should strengthen longitudinal, multilevel, and intervention‐based evidence to clarify how empowering work environments and leadership practices can support sustainable nursing workforces [36].

## 5. Implications for Nursing Management and Policy

These findings suggest that nursing workforce policy should move beyond strategies focused solely on increasing nurse supply. Although recruitment and education remain important, workforce sustainability also depends on whether nurses can practice in environments that provide adequate resources, professional autonomy, shared governance, leadership support, and opportunities for development. NWE and SE should therefore be considered policy‐relevant organizational targets rather than only local management concerns.

At the health system level, workforce policies should encourage organizations to monitor and improve practice environments as part of nurse retention and care quality strategies. Safe staffing, access to material and informational resources, supportive leadership, protection from workplace violence, and participation in decision‐making should be embedded into institutional workforce planning and quality improvement policies. SE provides a useful framework for translating these priorities into organizational reform because it links access to resources, support, information, opportunity, and decision‐making power with nurse well‐being and workforce outcomes. Policies that invest in nursing leadership development and empowering work environments may help reduce burnout, strengthen nurse retention, and improve care quality and patient safety.

## 6. Limitations

This study has several limitations. First, although WoSCC and Scopus were used to improve literature coverage, relevant publications indexed in other databases may have been missed, and the inclusion of only English‐language research articles may have introduced language and publication bias. Second, bibliometric indicators such as publication counts, citation counts, co‐citation links, burst keywords, and network centrality reflect visibility and knowledge structure, but they do not necessarily indicate methodological quality, clinical importance, or the strength of evidence. Third, despite data cleaning and harmonization, variations in author names, institutional names, journal titles, keywords, and cited references may not have been completely eliminated, and the interpretation of keyword clusters and thematic evolution inevitably involved some subjectivity. Finally, this study provides a cross‐sectional bibliometric overview based on records retrieved up to March 31, 2026; therefore, it can identify publication patterns, collaboration structures, influential literature, and thematic trends but cannot establish causal relationships or directly assess changes in evidence quality over time.

## 7. Conclusion

This bibliometric review shows that research on NWE and SE has evolved from measurement and outcome description toward mechanism‐oriented research on empowerment, leadership, and workforce sustainability. The findings indicate that nursing workforce challenges should be interpreted through a linked chain of organizational conditions: global workforce shortages are shaped not only by supply but also by practice environments, SE, leadership, and workforce outcomes. Future research should strengthen longitudinal and intervention‐based evidence. At the policy level, organizational reforms that improve practice environments, support SE, and develop nursing leadership should be considered essential strategies for sustaining the nursing workforce and improving care quality.

## Author Contributions

Study design: Xin Su, Mengnan Liu. Data collection: Yan Jiang, Han He. Data analysis: Yan Jiang, Haobo Wang, Xin Su, Mengnan Liu. Study supervision: Gang Luo, Jinwen Wu. Manuscript writing: Xin Su, Mengnan Liu. Critical revisions for important intellectual content: Xinyi He, Xinyi Fan, Yanqi Wu, Gang Luo, Jinwen Wu. Xin Su, Mengnan Liu, and Yan Jiang have contributed equally to this work and are regarded as the co‐first authors.

## Funding

This work was supported by the Sichuan Provincial Department of Science and Technology Project (2026NSFSC1823), Youth Innovation Project of Sichuan Medical Association (Q20250014), and Southwest Medical University Project (2024ZXYZX39, 2024ZKY073).

## Disclosure

All authors have read and agreed to the published version of the manuscript. The funder had no role in the study design, data analysis, or decision to publish.

## Ethics Statement

This bibliometric review of public data did not require ethics approval or informed consent.

## Conflicts of Interest

The authors declare no conflicts of interest.

## Supporting Information

Additional supporting information can be found online in the Supporting Information section.

## Supporting information


**Supporting Information 1** Supporting Table S1: Complete search strategies for WoSCC and Scopus.

**Supporting Information 2** Supporting Table S2: List of included records with DOI and title.

**Supporting Information 3** Supporting Table S3: Key parameters used in CiteSpace and VOSviewer analyses.

## Data Availability

The data are available on request from the authors.

## References

[1] Boniol M. , Kunjumen T. , Nair T. S. , Siyam A. , Campbell J. , and Diallo K. , The Global Health Workforce Stock and Distribution in 2020 and 2030: A Threat to Equity and ‘Universal’ Health Coverage?, BMJ Global Health. (2022) 7, no. 6, 10.1136/bmjgh-2022-009316. PMC9237893 35760437

[2] Ren H. , Li P. , Xue Y. , Xin W. , and Yin X. , Global Prevalence of Nurse Turnover Rates: A Meta-Analysis of 21 Studies From 14 Countries, Journal of Nursing Management. (2024) 2024, no. 1, 10.1155/2024/5063998. PMC11919231 40224762

[3] Heinen M. M. , Van Achterberg T. , Schwendimann R. et al., Nurses’ Intention to Leave Their Profession: A Cross Sectional Observational Study in 10 European Countries, International Journal of Nursing Studies. (2013) 50, no. 2, 174–184, 10.1016/j.ijnurstu.2012.09.019. 23107005

[4] White E. M. , Aiken L. H. , Sloane D. M. , and Mchugh M. D. , Nursing Home Work Environment, Care Quality, Registered Nurse Burnout and Job Dissatisfaction, Geriatric Nursing. (2020) 41, no. 2, 158–164, 10.1016/j.gerinurse.2019.08.007. 31488333 PMC7051884

[5] Bruyneel A. , Dauvergne J. E. , Bouckaert N. et al., Association of Burnout and Intention-to-Leave the Job With Objective Nursing Workload and Nursing Working Environment: A Cross-Sectional Study Among Intensive Care Nurses, Journal of Clinical Nursing. (2025) 34, no. 10, 4281–4292, 10.1111/jocn.17650. 39809579

[6] Kanter R. M. , Men and Women of the Corporation, 1977, Basic Books, New York.

[7] Kanter R. M. , Men and Women of the Corporation, 1993, 2nd edition, Basic Books, New York.

[8] Yesilbas H. and Kantek F. , Relationship Between Structural Empowerment and Job Satisfaction Among Nurses: A Meta-Analysis, International Nursing Review. (2024) 71, no. 3, 484–491, 10.1111/inr.12968. 38642048

[9] Orlowska A. and Laguna M. , Structural and Psychological Empowerment in Explaining Job Satisfaction and Burnout in Nurses: A Two-Level Investigation, Journal of Nursing Management. (2023) 2023, 9958842–11, 10.1155/2023/9958842. 40225690 PMC11918508

[10] Boamah S. A. , Spence Laschinger H. K. , Wong C. , and Clarke S. , Effect of Transformational Leadership on Job Satisfaction and Patient Safety Outcomes, Nursing Outlook. (2018) 66, no. 2, 180–189, 10.1016/j.outlook.2017.10.004. 29174629

[11] Valle R. , Balsanelli A. P. , Taminato M. , Saconato H. , and Gasparino R. , The Relationship Between the Authentic Leadership of Nurses and Structural Empowerment: A Systematic Review, Revista da Escola de Enfermagem da USP. (2021) 55, 10.1590/s1980-220x2019029003667. 33886899

[12] Wong C. A. and Laschinger H. K. , Authentic Leadership, Performance, and Job Satisfaction: The Mediating Role of Empowerment, Journal of Advanced Nursing. (2013) 69, no. 4, 947–959, 10.1111/j.1365-2648.2012.06089.x. 22764828

[13] Van Eck N. J. and Waltman L. , Software Survey: Vosviewer, a Computer Program for Bibliometric Mapping, Scientometrics. (2010) 84, no. 2, 523–538, 10.1007/s11192-009-0146-3. 20585380 PMC2883932

[14] Synnestvedt M. B. , Chen C. , and Holmes J. H. , Citespace II: Visualization and Knowledge Discovery in Bibliographic Databases, AMIA’s Annual Symposium. (2005) 2005, 724–728. PMC1560567 16779135

[15] Paguio J. T. , Yu D. S. F. , and Su J. J. , Systematic Review of Interventions to Improve Nurses’ Work Environments, Journal of Advanced Nursing. (2020) 76, no. 10, 2471–2493, 10.1111/jan.14462. 32770584

[16] Lake E. T. , Sanders J. , Duan R. , Riman K. A. , Schoenauer K. M. , and Chen Y. , A Meta-Analysis of the Associations Between the Nurse Work Environment in Hospitals and 4 Sets of Outcomes, Medical Care. (2019) 57, no. 5, 353–361, 10.1097/mlr.0000000000001109. 30908381 PMC6615025

[17] Chang Y. C. , Chang H. Y. , and Feng J. Y. , Appraisal and Evaluation of the Instruments Measuring the Nursing Work Environment: A Systematic Review, Journal of Nursing Management. (2022) 30, no. 3, 670–683, 10.1111/jonm.13559. 35146825

[18] Wagner J. I. , Cummings G. , Smith D. L. , Olson J. , Anderson L. , and Warren S. , The Relationship Between Structural Empowerment and Psychological Empowerment for Nurses: A Systematic Review, Journal of Nursing Management. (2010) 18, no. 4, 448–462, 10.1111/j.1365-2834.2010.01088.x. 20609049

[19] Cummings G. G. , Tate K. , Lee S. et al., Leadership Styles and Outcome Patterns for the Nursing Workforce and Work Environment: A Systematic Review, International Journal of Nursing Studies. (2018) 85, 19–60, 10.1016/j.ijnurstu.2018.04.016. 29807190

[20] Li L. Z. , Yang P. , Singer S. J. , Pfeffer J. , Mathur M. B. , and Shanafelt T. , Nurse Burnout and Patient Safety, Satisfaction, and Quality of Care: A Systematic Review and Meta-Analysis, JAMA Network Open. (2024) 7, no. 11, 10.1001/jamanetworkopen.2024.43059. PMC11539016 39499515

[21] Lake E. T. , Development of the Practice Environment Scale of the Nursing Work Index, Research in Nursing & Health. (2002) 25, no. 3, 176–188, 10.1002/nur.10032. 12015780

[22] Aiken L. H. , Cimiotti J. P. , Sloane D. M. et al., Effects of Nurse Staffing and Nurse Education on Patient Deaths in Hospitals With Different Nurse Work Environments, The Journal of Nursing Administration. (2012) 42, no. 10 Suppl, S10–S16, 10.1097/01.Nna.0000420390.87789.67. 22976889 PMC6764437

[23] Aiken L. H. , Sloane D. M. , Clarke S. et al., Importance of Work Environments on Hospital Outcomes in Nine Countries, International Journal for Quality in Health Care. (2011) 23, no. 4, 357–364, 10.1093/intqhc/mzr022. 21561979 PMC3136199

[24] Aiken L. H. , Sermeus W. , Van Den Heede K. et al., Patient Safety, Satisfaction, and Quality of Hospital Care: Cross Sectional Surveys of Nurses and Patients in 12 Countries in Europe and the United States, BMJ. (2012) 344, no. mar20 2, 10.1136/bmj.e1717. PMC3308724 22434089

[25] Laschinger H. K. , Finegan J. , Shamian J. , and Wilk P. , Impact of Structural and Psychological Empowerment on Job Strain in Nursing Work Settings: Expanding Kanter’s Model, Journal of Nursing Administration. (2001) 31, no. 5, 260–272, 10.1097/00005110-200105000-00006. 11388162

[26] Laschinger H. K. S. , Finegan J. E. , Shamian J. , and Wilk P. , A Longitudinal Analysis of the Impact of Workplace Empowerment on Work Satisfaction, Journal of Organizational Behavior. (2004) 25, no. 4, 527–545, 10.1002/job.256.

[27] Fragkos K. C. , Makrykosta P. , and Frangos C. C. , Structural Empowerment Is a Strong Predictor of Organizational Commitment in Nurses: A Systematic Review and Meta-Analysis, Journal of Advanced Nursing. (2020) 76, no. 4, 939–962, 10.1111/jan.14289. 31820471

[28] Boamah S. , Linking Nurses’ Clinical Leadership to Patient Care Quality: The Role of Transformational Leadership and Workplace Empowerment, Canadian Journal of Nursing Research. (2018) 50, no. 1, 9–19, 10.1177/0844562117732490. 29108423

[29] Aiken L. H. , Lasater K. B. , Sloane D. M. et al., Physician and Nurse Well-Being and Preferred Interventions to Address Burnout in Hospital Practice: Factors Associated With Turnover, Outcomes, and Patient Safety, JAMA Health Forum. (2023) 4, no. 7, 10.1001/jamahealthforum.2023.1809. PMC10329209 37418269

[30] Nantsupawat A. , Kunaviktikul W. , Nantsupawat R. , Wichaikhum O. , Thienthong H. , and Poghosyan L. , Effects of Nurse Work Environment on Job Dissatisfaction, Burnout, Intention to Leave, International Nursing Review. (2017) 64, no. 1, 91–98, 10.1111/inr.12342. 27882573

[31] White E. M. , Aiken L. H. , and Mchugh M. D. , Registered Nurse Burnout, Job Dissatisfaction, and Missed Care in Nursing Homes, Journal of the American Geriatrics Society. (2019) 67, no. 10, 2065–2071, 10.1111/jgs.16051. 31334567 PMC6800779

[32] Havaei F. , Ma A. , Staempfli S. , and Macphee M. , Nurses’ Workplace Conditions Impacting Their Mental Health During COVID-19: A Cross-Sectional Survey Study, Healthcare (Basel). (2021) 9, no. 1, 10.3390/healthcare9010084. PMC7830057 33467080

[33] Roche M. , Diers D. , Duffield C. , and Catling-Paull C. , Violence Toward Nurses, the Work Environment, and Patient Outcomes, Journal of Nursing Scholarship. (2010) 42, no. 1, 13–22, 10.1111/j.1547-5069.2009.01321.x. 20487182

[34] Bruyneel A. , Bouckaert N. , Maertens De Noordhout C. et al., Association of Burnout and Intention-to-Leave the Profession with Work Environment: A Nationwide Cross-Sectional Study Among Belgian Intensive Care Nurses After Two Years of Pandemic, International Journal of Nursing Studies. (2023) 137, 10.1016/j.ijnurstu.2022.104385. PMC9640385 36423423

[35] Sihvola S. , Nurmeksela A. , Mikkonen S. , Peltokoski J. , and Kvist T. , Resilience, Job Satisfaction, Intentions to Leave Nursing and Quality of Care Among Nurses During the COVID-19 Pandemic: A Questionnaire Study, BMC Health Services Research. (2023) 23, no. 1, 10.1186/s12913-023-09648-5. PMC10266309 37316918

[36] Gifford W. A. , Squires J. E. , Angus D. E. et al., Managerial Leadership for Research Use in Nursing and Allied Health Care Professions: A Systematic Review, Implementation Science. (2018) 13, no. 1, 10.1186/s13012-018-0817-7. PMC6161344 30261927

