# Supplementary material for: Global Research on Nursing Work Environment and Structural Empowerment: Trends and Thematic Evolution in Leadership and Workforce Outcomes—A Bibliometric Analysis
Source: J Nurs Manag. 2026 Sep 14;2026:6020647. doi: 10.1155/jonm/6020647 (PMC13575484; doi:10.1155/jonm/6020647)
Supplement: Supplementary file 1 [file JONM-2026-6020647-s001.docx]

**Supplementary Table S1.** Complete search strategies for WoSCC and Scopus.

**Database:** Web of Science Core Collection

**Search date:** 31 March 2026

**Field tag:** TS

**Search query:**

TS = (nurs* AND ("work environment" OR "practice environment" OR "nursing work environment" OR "practice environment scale" OR "structural empowerment" OR autonomy OR "shared governance"))

**Indexes:**

Science Citation Index Expanded and Social Sciences Citation Index

**Limits:**

- Language: English
- Document type: Article
- Timespan: 2007–2026

**Database:** Scopus

**Search date:** 31 March 2026

**Field tag:** TITLE-ABS-KEY

**Search query:**

TITLE-ABS-KEY ( nurs* AND ( "work environment" OR "practice environment" OR "nursing work environment" OR "practice environment scale" OR "structural empowerment" OR autonomy OR "shared governance" ) )

**Limits:**

- Language: English
- Document type: Article
- Timespan: 2007–2026
