# Supplementary material for: Global Research on Nursing Work Environment and Structural Empowerment: Trends and Thematic Evolution in Leadership and Workforce Outcomes—A Bibliometric Analysis
Source: J Nurs Manag. 2026 Sep 14;2026:6020647. doi: 10.1155/jonm/6020647 (PMC13575484; doi:10.1155/jonm/6020647)
Supplement: Supplementary file 2 [file JONM-2026-6020647-s003.docx]

**Supplementary Table S3.** Key parameters used in CiteSpace and VOSviewer analyses.

| **Figure/ analysis** | **Software** | **Network type/node type** | **Time slicing** | **Threshold/selection criteria** | **Counting/normalization /link strength** | **Pruning algorithm** | **Clustering algorithm** |
| --- | --- | --- | --- | --- | --- | --- | --- |
| Institutional collaboration network | VOSviewer | Co-authorship; unit: Organizations | Not applicable | Minimum number of documents: 5 | Full counting; association strength normalization | Not applicable | Default VOSviewer clustering algorithm based on the VOS clustering technique |
| Author collaboration network | VOSviewer | Co-authorship; unit of analysis: authors | Not applicable | Minimum number of documents of an author: 5 | Full counting; association strength normalization | Not applicable | Default VOSviewer clustering algorithm based on the VOS clustering technique |
| Co-cited author network | VOSviewer | Co-citation; unit of analysis: cited authors | Not applicable | Minimum number of citations of a cited author: 50 | Full counting; association strength normalization | Not applicable | Default VOSviewer clustering algorithm based on the VOS clustering technique |
| Journal network | VOSviewer | Citation / co-citation; unit of analysis: sources | Not applicable | Minimum number of documents or citations of a source: 5 | Full counting; association strength normalization | Not applicable | Default VOSviewer clustering algorithm based on the VOS clustering technique |
| Co-cited journal network | VOSviewer | Co-citation; unit of analysis: cited sources | Not applicable | Minimum number of citations of a cited source: 20 | Full counting; association strength normalization | Not applicable | Default VOSviewer clustering algorithm based on the VOS clustering technique |
| Dual-map overlay of journals | CiteSpace | Citing journals and cited journals | 2007–2026; 1 year per slice | g-index, k = 25 | Cosine; within slices | None | Not applicable |
| Timeline view of reference co-citation analysis | CiteSpace | Node type: reference | 2007–2026; 1 year per slice | g-index, k = 25 | Cosine; within slices | None | Default CiteSpace clustering; cluster labels generated by log-likelihood ratio (LLR) |
| Cluster view of reference co-citation analysis | CiteSpace | Node type: reference | 2007–2026; 1 year per slice | g-index, k = 25 | Cosine; within slices | None | Default CiteSpace clustering; cluster labels generated by log-likelihood ratio (LLR) |
| Top 25 references with the strongest citation bursts | CiteSpace | Node type: reference | 2007–2026; 1 year per slice | g-index, k = 25; burst detection | Cosine; within slices | Not applicable | Not applicable |
| Keyword density map | VOSviewer | Co-occurrence; unit of analysis: all keywords | Not applicable | Minimum number of occurrences of a keyword: 30 | Full counting; association strength normalization | Not applicable | Default VOSviewer clustering algorithm based on the VOS clustering technique |
| Timeline view of keyword analysis | CiteSpace | Node type: keyword | 2007–2026; 1 year per slice | g-index, k = 25 | Cosine; within slices | None | Default CiteSpace clustering; cluster labels generated by log-likelihood ratio (LLR) |
| Cluster view of keyword analysis | CiteSpace | Node type: keyword | 2007–2026; 1 year per slice | g-index, k = 25 | Cosine; within slices | None | Default CiteSpace clustering; cluster labels generated by log-likelihood ratio (LLR) |
| Top 25 keywords with the strongest citation bursts | CiteSpace | Node type: keyword | 2007–2026; 1 year per slice | g-index, k = 25; burst detection | Cosine; within slices | Not applicable | Not applicable |

Note: For CiteSpace analyses, time slicing was set from 2007 to 2026, with one year per slice. The g-index with k = 25 was used as the selection criterion. Link strength was calculated using cosine similarity within slices. No pruning algorithm was selected unless otherwise stated. Cluster labels were generated using the default log-likelihood ratio (LLR) algorithm. For VOSviewer analyses, full counting and association strength normalization were used. Clusters were generated using the default VOSviewer clustering algorithm based on the VOS clustering technique. Specific thresholds were set according to the corresponding analysis type and are reported in the table.
